# A Systematic Review of Federated and Cloud Computing Approaches for Predicting Mental Health Risks

**DOI:** 10.3390/s26010229

**Published:** 2025-12-30

**Authors:** Iram Fiaz, Nadia Kanwal, Amro Al-Said Ahmad

**Affiliations:** School of Computer Science and Mathematics, Keele University, Newcastle ST5 5BG, UK; n.kanwal@keele.ac.uk (N.K.); a.m.al-said.ahmad@keele.ac.uk (A.A.-S.A.)

**Keywords:** mental health, federated learning, cloud computing, edge computing, privacy preserving, machine learning

## Abstract

Mental health disorders affect large numbers of people worldwide and are a major cause of long-term disability. Digital health technologies such as mobile apps and wearable devices now generate rich behavioural data that could support earlier detection and more personalised care. However, these data are highly sensitive and distributed across devices and platforms, which makes privacy protection and scalable analysis challenging; federated learning offers a way to train models across devices while keeping raw data local. When combined with edge, fog, or cloud computing, federated learning offers a way to support near-real-time mental health analysis while keeping raw data local. This review screened 1104 records, assessed 31 full-text articles using a five-question quality checklist, and retained 17 empirical studies that achieved a score of at least 7/10 for synthesis. The included studies were compared in terms of their FL and edge/cloud architectures, data sources, privacy and security techniques, and evidence for operation in real-world settings. The synthesis highlights innovative but fragmented progress, with limited work on comorbidity modelling, deployment evaluation, and common benchmarks, and identifies priorities for the development of scalable, practical, and ethically robust FL systems for digital mental health.

## 1. Introduction

Mental health disorders continue to pose a significant and expanding challenge to global health, with current estimates suggesting that around 970 million individuals are affected and that these conditions account for more than 14% of the total years lived with disability worldwide (WHO, 2022 [1]; Kestel et al., 2022 [2]). Although awareness of mental health needs has increased, access to timely, personalised, and ethically delivered care remains inadequate, especially in settings with limited resources and in communities with restricted digital access.

The rapid growth of digital health solutions has created new opportunities for detection, ongoing monitoring, and more tailored support. Nevertheless, the benefits of these developments are limited by the sensitive nature of mental health information, which is frequently dispersed across mobile phones, wearable devices, and clinical platforms. This fragmentation creates complications for data sharing, reliable integration, and consistent clinical applicability (Karagarandehkordi et al., 2025 [3]).

Federated learning provides a privacy-aware alternative to traditional machine learning because it allows models to be trained across separate data sources without the need to move raw information to a central server (McMahan et al., 2017) [4]. This approach aligns naturally with the distributed and varied nature of data found in mental health settings. When paired with edge and cloud-based computing, federated learning systems can offer real-time inference, reduce communication demands, and give users greater control over their personal information [5]. Despite these advantages, practical adoption of federated learning within mental health remains limited. Questions surrounding scalability, inclusivity across different diagnostic groups, and the strength of privacy protections are still largely unanswered (Dubey et al., 2025) [6].

Although earlier reviews have explored federated learning in the broader healthcare domain (Zhou et al., 2021 [7]; Dhade & Shirke, 2024 [8]), few have specifically addressed the distinctive clinical, technical, and ethical issues that arise in mental health contexts. More recent reviews focused on this area (Khalil et al., 2024 [9]; Grataloup & Kurpicz-Briki, 2024 [10]) highlight encouraging use cases but also reveal gaps in realistic deployment, the modelling of comorbid conditions, and the integration of multiple data types.

To address these gaps, this review synthesises 17 empirical studies that apply FL in mental health settings with explicit integration of edge, fog, or cloud computing. All candidate studies were evaluated using a structured five-question quality checklist, and only those scoring at least 7/10 were retained for detailed synthesis (see Section 3.4 and Appendix A.1 and Appendix A.2). This study is guided by four research questions:

RQ1: How have federated learning, cloud, and edge computing been implemented and evaluated in mental health systems?
  Rationale: Examining the strategies used to design and assess these systems is essential for determining whether federated learning, combined with cloud and edge computing, can be applied effectively and reliably in real-world mental health environments.
RQ2: How diverse is the data used to predict mental health risks?
  Rationale: Understanding the range of data sources, including demographic, clinical, and behavioural information, is important for assessing how data heterogeneity influences model generalisation and predictive accuracy.
RQ3: What privacy and security techniques are adopted across FL frameworks?
  Rationale: Evaluating the privacy-preserving methods used in these systems helps determine whether strong confidentiality can be maintained while still achieving reliable predictive performance in sensitive mental health settings.
RQ4: What challenges and limitations do studies report regarding scalability, evaluation, and deployment?
  Rationale: Identifying barriers such as technical limitations, regulatory considerations, and diagnostic constraints is relevant to understanding the practical readiness to develop these systems, in addition to providing insight into areas where further development is needed to support secure and scalable mental health prediction

By examining these areas in detail, the aim of the review is to guide the development of federated learning systems that are scalable, respectful of privacy, and meaningful for clinical use in digital mental health. The remainder of the paper is structures as follows. Section 2 outlines the research background and related literature. Section 3 describes the methodology and the search process. Section 4 summarises the included studies, followed by a synthesis of the main observations in Section 5. Section 6 presents the findings in relation to the research questions. Section 7 discusses their implications and outlines directions for future research.

## 2. Background and Related Work

### 2.1. Federated Learning in Mental Health AI

Federated learning (FL) enables collaborative model training across decentralised clients while preserving data locality. Originally proposed by McMahan et al. in 2017 [4], FL has gained traction in health care due to its alignment with privacy regulations and distributed data ecosystems. In mental health, where data is often sparse, non-IID, and multimodal, FL offers a compelling alternative to centralised learning (Kairouz et al., 2021) [11].

Recent reviews (Khalil et al., 2024 [9]; Grataloup & Kurpicz-Briki, 2024 [10]) show that FL has been applied to tasks such as depression detection, seizure monitoring, and emotion recognition. However, most studies rely on small-scale or synthetic datasets, and few address challenges such as client dropout, fairness, or comorbidity-aware modelling. FedAvg remains the dominant aggregation algorithm, despite known limitations in heterogeneous environments [11].

### 2.2. Role of Edge and Cloud Computing

FL systems rely on underlying infrastructure that coordinates training, manages communication, and promotes scalability. Cloud computing offers high-throughput coordination and storage, while edge computing provides local inference and low-latency responsiveness [12]. Fog computing, located between the two, can cache data and reduce bandwidth pressure [13].

In mental health contexts, edge–cloud integration is theorized to align well with FL; however, empirical validation remains limited. A recent literature review by Karamthulla et al. [14] discovered that despite the growing use of AI-powered cloud systems in diagnostics and monitoring, the limited literature compares latency, energy consumption, and fault tolerance of deployed applications. On the same note, the World Health Organization Regional Office for Europe [15] states that a large number of AI models within the mental health domain are promoted without sufficient evaluation of their infrastructural viability.

### 2.3. Existing Reviews and Gaps

A number of reviews have analysed FL in healthcare overall [16,17], but very few have looked at the diagnostic, infrastructural, and ethical nuances of mental health. Khalil et al. [9] conducted the first systematic review dedicated to FL in mental health, identifying 27 studies but noting that most lacked deployment realism and multimodal integration. Grataloup and Kurpicz-Briki [10] similarly found that while FL is conceptually aligned with mental-state detection, empirical validation remains sparse.

Other reviews [15,18] have made the critique that AI-based mental health research has methodological shortcomings, such as inadequate transparency and insufficient data diversity and reproducibility. These gaps undermine the development of clinically viable, ethically sound FL systems.

A further underexplored area is privacy and security validation. While FL is often assumed to be inherently privacy-preserving due to its decentralised structure, several foundational studies [11,19] caution that without formal mechanisms, such as differential privacy (DP), secure multi-party computation (SMPC), or communication encryption, privacy guarantees may be incomplete or misleading. Research focusing on FL security [20,21] mention that most healthcare deployments lack threat modelling, cryptographic protection, or comparative evaluations of overhead and risk exposure. However, not many mental health-specific studies involve such methods, even though behavioural and psychiatric data are more sensitive. The lack of internalized privacy measures and empirical security testing restricts the interpretation and credibility of FL systems in clinical fields.

Earlier reviews of FL in healthcare have mostly taken a broad clinical or technical view, cataloguing application domains, model types, and high-level privacy motivations across general health care rather than examining the infrastructural and diagnostic specifics of mental health systems [16,17]. In contrast, the mental health-focused FL reviews by Khalil et al. and Grataloup and Kurpicz-Briki primarily summarise use cases such as depression and mental state detection, with relatively limited analysis of concrete FL architectures, edge/cloud/fog deployment patterns, or the empirical evaluation of privacy and security mechanisms [9,10]. Building on these contributions, the present review systematically maps how FL, cloud, edge, and fog components are architected and deployed in mental health applications; analyses diagnostic and data-modality diversity (including comorbid scenarios); and provides a study-level inventory of implemented privacy and security techniques, together with their reported overheads and implications for deployment realism.

### 2.4. Scope and Contribution

Building on the collective research efforts outlined in Section 2.3, this review focuses specifically on empirical studies that integrate FL with cloud, edge, or fog computing for mental health applications. It considers a limited set of studies that report quantitative performance measures and treat mental health conditions as a primary application.

There are four key contributions that are highlighted in this review:

1. **Architectural Mapping:** Describing how FL, cloud, and edge components are implemented and evaluated in practice (RQ1);
2. **Data Diversity Analysis:** Assessing the diversity of mental health conditions, data modalities, and comorbidity modelling strategies (RQ2).
3. **Privacy and Security Review:** Examining the application of differential privacy, encryption, and access-control mechanisms (RQ3);
4. **Limitation Synthesis:** Identifying recurring methodological and infrastructural challenges (RQ4).

This review seeks to inform the design of digital mental health FL systems that are scalable, privacy-conscious, and clinically relevant by synthesising these dimensions.

## 3. Systematic Literature Review Methodology

**Figure 1:**
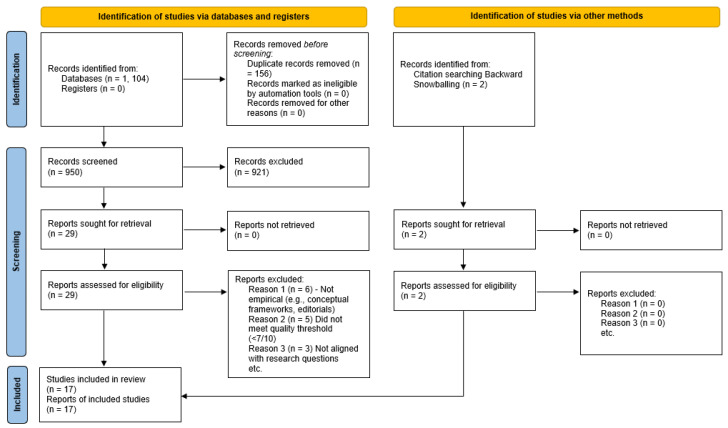
PRISMA workflow (Based on Page MJ, et al. [22]).

This systematic review was conducted to critically synthesise empirical research investigating the integration of federated learning (FL), edge/cloud computing, and privacy-preserving AI within mental health contexts. The review methodology followed the PRISMA 2020 framework [22] to ensure a transparent and reproducible workflow, from literature identification through selection, assessment, and synthesis. The PRISMA diagram illustrating the screening process is shown in Figure 1. All the PRISMA checklists and workflow processes (following guidelines from [22]) are available as Supplementary Materials to ensure transparency and reproducibility.

In addition, the review was designed and conducted in accordance with the systematic literature review procedures outlined by Kitchenham and Charters [23], who provide structured guidance for planning, executing, and reporting SLRs in software engineering and computing research.

### 3.1. Search Strategy

Automated and manual search strategies were used to capture relevant empirical studies. The automated search was run on five major academic databases (ACM Digital Library, IEEE Xplore, ScienceDirect, SpringerLink, and Scopus). The Boolean string combined four core domains—mental health, FL, edge/cloud computing, and privacy/security—and was applied to titles, abstracts, and keywords:

(“mental health” OR “depression” OR “anxiety”) AND (“federated learning”) AND (“edge computing” OR “cloud computing”)

Although the string foregrounded ‘mental health’, ‘depression’, and ‘anxiety’, several of the final studies also focused on related or comorbid conditions such as stress, epilepsy, Alzheimer’s disease, autism, and chronic disease.These papers entered the pool in two ways: (i) through database records where these conditions were explicitly framed as part of mental health monitoring or neurological decline in conjunction with edge or cloud deployment and (ii) via backward snowballing from the reference lists of FL–mental health–edge/cloud articles retained at the full-text stage.

This reduced reliance on the initial diagnostic keywords alone and helped surface work where mental health was described more broadly (e.g., ‘stress’, ‘cognitive decline’, and ‘chronic disease’) and where cloud, fog, or edge infrastructure was described in the methods rather than in the title.

Privacy and security were treated as conceptually important but were not added to the Boolean string to avoid overly restrictive filtering at the retrieval stage. Instead, these aspects were captured through full-text screening and data extraction. The automated search was complemented by a backward snowballing procedure in which the reference lists of all full-text articles were checked for additional eligible studies [24,25]. Nonetheless, there remains a residual risk that FL studies involving mental health-relevant conditions or edge/fog deployments but lacking explicit mental health or edge/cloud terminology in titles or abstracts were missed; this limitation is considered when interpreting the scope of the review.

### 3.2. Screening and Selection Process

The initial search retrieved 1021 unique records, which were imported into the Rayyan AI platform for duplicate removal and screening [26]. In Phase 1, titles, abstracts, and keywords were screened by the lead author for relevance to the research questions, resulting in the exclusion of 992 records and leaving 29 studies for full-text review. To improve coverage, backward snowballing was then applied to the reference lists of these 29 papers, yielding two additional studies that met the preliminary criteria and bringing the full text pool to 31 articles.

In Phase 2, all full texts were assessed against the inclusion and exclusion criteria. Screening and quality-assessment decisions were made by the lead reviewer and checked by two academic supervisors (second and third authors). A total of 14 studies were excluded for methodological or topical reasons, resulting in 17 empirical studies being retained for final synthesis.

### 3.3. Inclusion and Exclusion Criteria

*Inclusion Criteria*
  - Peer-reviewed journal articles, conference proceedings, or scholarly book chapters;
  - Empirical studies such as experiments, case studies, simulations, feasibility trials, or evaluations;
  - Studies explicitly addressing one or more of the predefined research questions;
  - Full-text articles written in English;
  - Articles published up to January 2025.
*Exclusion Criteria*
  - Secondary studies, meta-analyses, opinion pieces, editorials, or responses;
  - Books or non-peer-reviewed literature;
  - Publications not addressing federated learning, mental health, or edge/cloud deployment;
  - Studies that failed to meet the quality assessment threshold (see Section 3.4), which was developed by the authors based on Kitchenham and Charters’ guidelines for systematic reviews in software engineering [23].

### 3.4. Quality Assessment

To ensure methodological rigour, each of the 31 full-text studies was evaluated using a structured five-question evaluation checklist developed by the authors in accordance with Kitchenham and Charters’ guidelines [23]. The checklist was designed to capture both relevance and quality in the context of FL for mental health. Each question was scored as 2 (fully addressed), 1 (partially addressed), or 0 (not addressed):

1. Are the aims or objectives of the research clearly stated and relevant to mental health AI?
2. Is federated learning implemented or proposed, and are privacy or security concerns explicitly discussed?
3. Is cloud or edge computing integrated into the system for data processing, model training, or deployment?
4. Do the privacy or security techniques used directly contribute to the research goals (e.g., secure AI for mental health or privacy-preserving monitoring)?
5. Is the experimental setup (e.g., dataset, sample size, and performance metrics) clearly described and appropriate for mental health AI?

Studies receiving a cumulative score below 7 out of 10 were excluded. Following this quality assessment, 17 studies met the inclusion threshold and were selected for final synthesis. All studies were initially scored by the lead reviewer using this five-question checklist. A random subset of studies was independently assessed by the third author, and all scores and inclusion decisions were then reviewed by the second and third authors. Any uncertainties or borderline cases were resolved through discussion, so no study was excluded solely on the basis of a single reviewer’s judgement. A custom checklist was adopted because existing formal risk-of-bias tools are primarily designed for clinical or epidemiological trials and are less suited to hybrid FL and systems/ML studies, which means that our approach is less standardized than those instruments and relies partly on subjective judgement. The detailed quality assessment for all 31 full-text studies, including Q1–Q5 scores, total score, and inclusion or exclusion rationale, is provided in Appendix A.1 and Appendix A.2.

### 3.5. Data Extraction

A structured data extraction template was developed to ensure consistency in all 17 included studies. The lead reviewer (first author) extracted all data, with verification provided by supervisory academic staff (second and third authors). Extracted data fields included the following:

*Study Characteristics*
  - Title, author names, publication year, publication venue, abstract, and keywords.
*Study Design*
  - Empirical format (e.g., experiment, simulation, case study, and feasibility analysis).
*Methodological Features*
  - FL implementation (architectures, aggregation algorithms, and toolkits);
  - AI models used (e.g., CNN, LSTM, or Transformer).
  - Privacy or security methods (e.g., differential privacy, homomorphic encryption, or SMPC).
  - Deployment setting (cloud, edge, or fog) and hardware specifications;
  - Data characteristics (source type, data modality, and real-time/synthetic/public).
*Outcomes and Evaluation*
  - Model performance (accuracy, F1 score, and convergence metrics);
  - System-level evaluation (e.g., communication cost, latency, and energy efficiency);
  - Validation methods (e.g., cross-validation or real-time pilot testing);
  - Descriptive or quantitative reporting of privacy–performance trade-offs.

This structured process enabled thematic coding and comparative synthesis across studies to address the four core research questions.

### 3.6. Threats to Validity

This review has several limitations that should be acknowledged. First, the database search relied on specific keyword combinations in English, so FL studies involving mental health-relevant conditions or edge/fog deployments that used different terminology or appeared in non-English venues may have been missed, even though backward snowballing reduced this risk. Second, screening, quality assessment, and data extraction were led by a single reviewer, with verification by two supervisors, which may have introduced some selection or interpretation bias despite the use of a structured checklist. Third, substantial heterogeneity in study designs, datasets, and evaluation protocols limits direct comparability and precludes meta-analysis, so the synthesis emphasizes qualitative patterns rather than pooled quantitative effects. To address this limitation, the snowballing method was used at the end of the second stage of the selection process in order to find more primary studies in case some studies were missed during the search. This approach involved reviewing the reference lists of all included studies and identifying additional relevant papers cited within them, thereby ensuring a more comprehensive coverage of the literature and reducing the risk of overlooking relevant papers.

## 4. Overview of Included Studies

**Figure 2:**
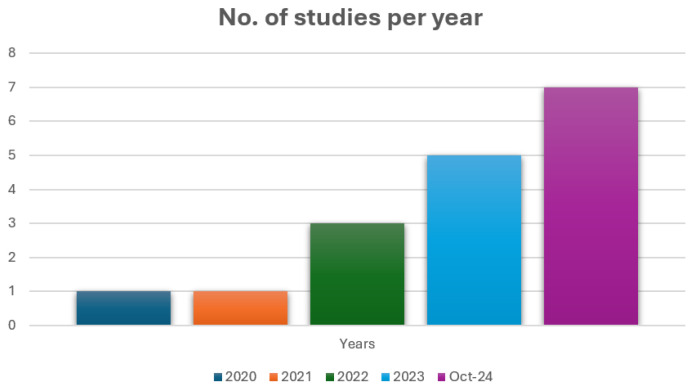
Number of included studies per year, showing the temporal trend in federated learning applications for mental health.

The 17 studies included in this review were published between 2021 and 2024, illustrating a rapidly evolving research focus on federated learning (FL) in mental health. The overview of studies is highlighted in Table 1. As shown in Figure 2, only two studies were published in 2021, with this number gradually increasing through 2022 and 2023, followed by a sharp rise to seven publications in the first ten months of 2024 alone. This pattern suggests growing recognition of FL as a viable privacy-preserving approach for distributed mental health modelling, particularly in the wake of increased global attention to mental well-being, decentralised health infrastructure, and data sensitivity in digital health applications (Dubey et al., 2025) [6].

**Figure 3:**
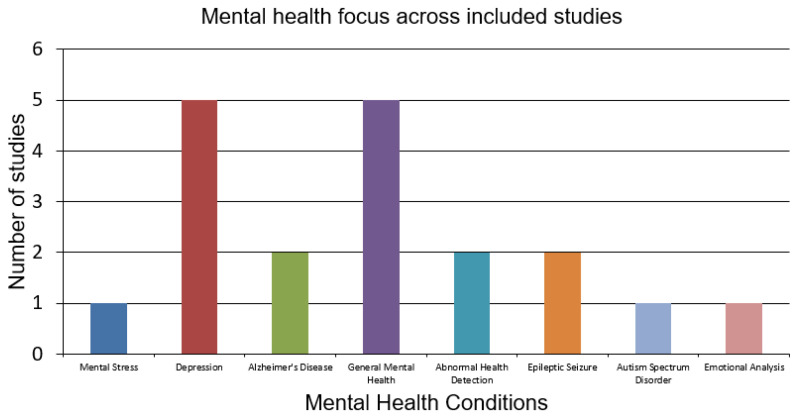
Distribution of mental health conditions across the 17 studies, highlighting the diagnostic focus of current federated learning research.

Thematically, the studies span a range of mental health conditions, though with a clear imbalance. Figure 3 highlights this distribution. Depression detection is the most frequently studied condition, addressed in five studies [27,28,29,30,31], typically leveraging social media, linguistic data, or smartphone behaviour. This focus corresponds with other recent observations by Jlassi et al. (2025) [32] and Ebrahimi et al. (2024) [33], who emphasised that depression research dominates FL applications due to the abundance of accessible, annotated datasets. General mental health monitoring, through activity recognition, stress detection, or behavioural proxy, is also prominent, appearing in another five studies [34,35,36,37,38].

The included studiesoften combine passive sensor streams and real-time inference from edge devices, aligned with the methodological practices described by Rashmi et al. (2023) [39]. Abnormal health detection (e.g., stroke or cognitive decline) appears in two studies [40,41], and epilepsy is addressed in two others [42,43]. Less frequently, FL is applied to Alzheimer’s disease [39,44], emotion analysis [38], and autism spectrum disorder [45]. Although several papers reference co-occurring physical or neurological conditions, such as brain tumours [39], asthma and stroke [40,41], or neurodegeneration [44], they do not explicitly model comorbidities in their frameworks. As Suruliraj and Orji (2022) [27] and Park D et al. (2024) [46] argue, FL for mental health has largely remained focused on one disease, limiting its relevance to the complex multimorbidity profiles seen in clinical practice.

**Table 1:** Overview of included studies.

Study	Ref.	Year	Mental Health Focus	Co-Existing Condition
(Alahmadi et al., 2024)	[34]	2024	Mental stress detection	–
(Suruliraj & Orji, 2022)	[27]	2022	Depression detection	–
(Rashmi et al., 2023)	[39]	2023	Alzheimer’s disease diagnosis (early stages)	Brain tumour
(Shaik et al., 2022)	[35]	2022	General mental health (remote patient monitoring)	–
(C. Zhang et al., 2024)	[36]	2024	General mental health (activity recognition)	–
(Nurmi et al., 2023)	[37]	2023	General mental health (chronic disease monitoring)	Diabetes, obesity, and respiratory diseases
(Ching et al., 2024)	[40]	2024	Abnormal health detection (depression, stroke)	Stroke and asthma (not analysed)
(Liu, 2024)	[28]	2024	Depression detection (social media-based FL)	Workplace depression (not analysed)
(Suryakala et al., 2024)	[42]	2024	Epilepsy seizure detection	Epilepsy-related comorbidities mentioned
(D. Y. Zhang et al., 2021)	[41]	2021	Abnormal health detection (depression and stroke)	Asthma (not analysed)
(Tabassum et al., 2023)	[29]	2023	Depression detection	Workplace depression (not analysed)
(Lakhan et al., 2023)	[45]	2023	Autism spectrum disorder detection	–
(Xu et al., 2022)	[30]	2022	Depression detection	–
(Chhikara et al., 2021)	[38]	2021	Emotion analysis (workplace stress and post-pandemic mental health)	Workplace stress (not analysed separately)
(Mandawkar & Diwan, 2024)	[44]	2024	Alzheimer’s disease detection	Neurodegenerative disorders (not analysed separately)
(Baghersalimi et al., 2024)	[43]	2024	Epileptic seizure detection	–
(Li et al., 2023)	[31]	2023	Depression detection	–

## 5. Synthesis and Observations

The temporal and diagnostic trends across the corpus are summarised in Figure 2 and Figure 3, which highlight both the recent growth in publications and the dominance of depression and general mental health monitoring among the included studies. The increasing number of publications over time signals growing momentum and research maturity. However, the diagnostic focus remains uneven. Depression and general behavioural mental health dominate the landscape, accounting for the majority of studies. This clustering highlights the reliance on accessible digital data streams, such as text, voice, and activity logs, which are well suited to FL architectures but represent only part of the mental health spectrum [28,29].

In contrast, conditions such as epilepsy, Alzheimer’s disease, autism, and emotional regulation disorders are under-represented, despite their clinical importance. Moreover, although several studies reference co-occurring physical or neurological conditions, none formally models comorbidities. This narrow diagnostic framing limits the generalisability and clinical relevance of current FL research, as multimorbidity is a defining feature of mental health populations in the real world [10,17].

The next section presents the detailed findings structured around the four research questions.

## 6. Findings Structured by Research Questions

### 6.1. RQ1: How Have Federated Learning, Cloud, and Edge Computing Been Implemented and Evaluated?

This research question examines how the 17 included studies operationalise FL in terms of architectural design, cloud–fog–edge integration, and evaluation practices.

#### 6.1.1. Federated Learning Architectures and Algorithms

Across the 17 studies, FL is predominantly implemented as cloud-centred horizontal FedAvg, with a central server coordinating updates from smartphones or IoT clients [27,29,30,31,35]. A smaller number of studies explore more advanced architectures, including hierarchical or multi-level aggregation, decentralised overlays, and edge-native deployments [36,39,40,41,43,44], but these remain exceptions rather than dominant design choices.

From a modelling perspective, most systems employ CNNs or shallow deep learning architectures, sometimes combined with sequence models or ensembles for multimodal fusion. For example, Lakhan et al. [45] use a federated CNN–LSTM pipeline for autism detection across fog–cloud infrastructure, while Chhikara et al. [38] combine CNN-based facial analysis with ensemble speech models for emotion recognition. Only one study proposes an explicitly asynchronous FL variant (CAFed) with differential-privacy noise to mitigate communication overhead and support partial client participation, but it remains centrally aggregated and is not extensively benchmarked against alternative coordination schemes [31]. Similar concerns about the joint optimisation of convergence and privacy under non-IID, delay-prone conditions are raised in broader FL work on hybrid optimisation strategies [47]. The findings across the FL methods are highlighted in Table 2.

**Table 2:** Federated learning architectures and algorithms.

Study	Type of Architecture	Algorithms Used in Federated Environment
[34]	Cloud server with FedAvg aggregation method	LDA, ANN, CNN
[27]	Cloud server with FedAvg aggregation method	Statistical anomaly detection
[39]	Cloud-based BrainCrossFed architecture	CNN and DINOv2
[35]	Cloud server with FedStack aggregation method	ANN, CNN, and Bi-LSTM
[36]	Multi-level FL (cloud and edge hierarchy)	TrMIFed, AsMIFed, and DmMIFed
[37]	Cloud-based FL	CNN
[40]	Decentralized FL with peer-to-peer overlay	DHT lookup, Bandit routing, and P2P aggregation
[28]	Federated deep learning (FDL) with global aggregation server	FedAPLF, XLM-RoBERTa, and TextGCN
[42]	Decentralized FL using Flower framework	Decision tree, MLP, and logistic regression
[41]	Edge-based FL	FedSense, EIDR, and AGUC
[29]	Cloud server with FedAvg	DL4J
[45]	Fog–cloud infrastructure with FedAvg	FCNN-LSTM
[30]	Cloud server with FedAvg	DeepMood (DMVM, DFM, and DNN)
[38]	Cloud server with FedAvg	CNN and ensemble ML classifiers
[44]	Blockchain-enabled cloud with FedAvg	TF-FedDeepCNN and ensemble CNN
[43]	P2P decentralized FL	Ensemble learning and knowledge distillation
[31]	Cloud-based asynchronous FL (FedAvg variant)	CAFed (CNN-based asynchronous FL with DP)

#### 6.1.2. Cloud and Edge Integration

Most studies adopt either edge–cloud or edge–fog–cloud deployment patterns, in which training is carried out on resource-constrained devices such as smartphones, wearables, or residential sensors, while model aggregation occurs on cloud servers, sometimes via intermediate fog nodes [27,29,30,34,35,36,39,40,45]. Fog layers are typically introduced as conceptual intermediaries to offload computation and communication, but very few studies provide empirical measurements of fog performance (e.g., latency, resilience, or failover). Many deployments are implemented as software-only simulations or small device pilots, and these configurations do not report behaviour under realistic network variability or client churn. The findings across cloud and edge deployments are highlighted in Table 3.

Communication and security mechanisms vary across deployments. Consumer-oriented systems tend to rely on Bluetooth and Wi-Fi links between sensors, smartphones, and cloud servers [27,34], whereas other studies incorporate encrypted TCP/IP channels, AES-secured wireless connections, or blockchain-based logging to strengthen confidentiality and auditability [30,31,38,39,44,45,48].

**Table 3:** Cloud and edge deployments.

Study	Deployment Level	Deployment Type	Client	Server	Communication Method
[34]	Edge–Fog–Cloud	Wearable sensors	Smartphone (simulated)	AWS Cloud	Bluetooth and Wi-Fi
[27]	Edge–Cloud	FL for smartphone MH sensing	Smartphone-based	AWS private cloud	Wi-Fi
[39]	Edge–Cloud	Cloud-assisted MRI	Hospital MRI workstations	Cloud (simulated)	Encrypted Internet communication (TCP/IP)
[35]	Edge–Cloud	Cloud-assisted remote patient monitoring	Wearable/IoT patient sensors	Cloud (simulated)	Wireless IoT transmission (Edge–Cloud)
[36]	Edge–Cloud	Multi-level FL (MIFed)	IoT edge clients and local servers	Global server aggregation	Wireless IoT data transfer with asynchronous updates
[37]	Edge–Cloud	FL with Local Differential Privacy (LDP-FL)	Radar, infrared, acoustic arrays, and depth cameras	Cloud (simulated)	Wireless IoT communication
[40]	Edge–Fog–Cloud	Decentralized FL using peer-to-peer (P2P) overlay architecture	Edge (cloud-based simulation)	Peer (Amazon EC2)	Wireless IoT communication between peers
[28]	Edge–Cloud	FL for social media-based depression detection	Social-media edge servers	Cloud (simulated)	Encrypted Internet-based model updates (secure server communication)
[42]	Edge–Cloud	FL for EEG-based seizure detection	Real EEG devices	Cloud	Secure wireless transmission (model updates)
[41]	Edge–Cloud	FL for abnormal health detection	Nvidia Jetson TX2/TX1/TK1 (wearable sensors)	Cloud (simulated)	Wireless IoT transmission (Wearable–Edge–Cloud)
[29]	Edge–Cloud	FL with smartphone sensing and cloud-assisted aggregation	Android devices	Cloud	Wireless IoT transmission
[45]	Edge–Fog–Cloud	FL for ASD detection	Edge–Fog (ASD lab)	Cloud	Secure AES-based model transmission
[30]	Edge–Cloud	FL with multi-source health data	Smartphones, keyboard sensors, and accelerometers	Cloud	AES encryption for model updates
[38]	Edge–Cloud	FL emotion recognition	IoT devices, facial expression, and speech recognition systems	Cloud	Encrypted transmission (model updates)
[44]	Edge–Cloud	Blockchain-enabled FL for Alzheimer detection	Medical sensors	Cloud	Encrypted ledger-based model updates
[43]	Edge	FL with adaptive ensemble learning	Edge across hospitals	Peer	Encrypted secure model updates
[31]	Edge–Cloud	Asynchronous FL (CAFed) for depression detection	Edge Weibo data (simulated)	Cloud	Secure model updates via differential privacy

#### 6.1.3. Evaluation Environments

Across the reviewed studies, evaluation typically reports both conventional predictive performance and system-level behaviour. Several works report very high classification scores in controlled or simulated settings (often ≥99% accuracy), for example, in BrainCrossFed and ASD detection [28,39,45], while studies using real, heterogeneous user data achieve more modest performance (e.g., 65% accuracy and 47% F1 score on 145 devices in [28]). The findings across federated learning and machine learning metrics used for evaluations are highlighted in Table 4.

System-level metrics that are critical for practical deployment, such as communication volume, latency, and energy consumption—are reported only sporadically. A small subset of studies quantifies bandwidth or power usage, showing, for example, that lightweight models and device preprocessing can drastically reduce update sizes and energy demand on mobile or embedded hardware [27,34,41,43], aligning with calls for energy-conscious, resource-aware FL design in health-critical edge environments [49]. However, most studies rely on simulations or small prototypes without consistent reporting of per round latency, end-to-end response time, or bandwidth cost, which limits cross-study comparability and obscures real-world feasibility.

Evaluation under non-IID and dynamically changing data distributions is also rare. One study explicitly demonstrates a 10–15% accuracy drop when moving from IID to non-IID settings [30], yet many others either assume uniform data or do not disclose the distributional structure, despite the fact that mobile, social media, and wearable data in mental health are inherently skewed and sparse. This gap contrasts with broader FL work that stresses the need to measure and stress test systems under realistic heterogeneity [28,50].

**Table 4:** FL and ML metrics across studies.

Study	Nodes	Rounds	Aggregation/FL Method	Model Update Frequency	ML Accuracy	Other ML Metrics
[34]	1000	1000	FedAvg	–	–	–
[27]	2	Multiple	Anomaly detection, FL scheduling	Periodic local	–	–
[39]	2	Multiple	FedAvg	Every round	99.77% (BCF)	F1: 100%, Precision: 100%
[35]	10	Multiple	Fedstack	Every round	ANN: 98%, CNN: 99%, Bi-LSTM: 93%	–
[36]	20	50	TrMLFed, AsMLFed, DmMLFed	Every round	92%	–
[37]	500	DHT-based, P2P overlay	P2P overlay every round	Every round	ResNet-34: 53%, ShuffleNet: 75.5%	–
[28]	145	Up to 70	FedAvg	One epoch per device	65%	F1: 47%, Precision: 69%, Recall: 46%
[42]	5	100	FedAvg (Flower)	Every round	MLP: 99%, DT: 94%, LR: 89%	Sensitivity: 98.24%, Specificity: 99.23%
[41]	30	Up to 200	Fedsens adaptive (local/global)	Every K rounds	Drowsiness: 81.6%, Stress: 8%	F1 (drowsiness): 0.816; F1 (stress): 0.08
[29]	5	5	FedAvg (Firebase DL4J)	Every round	68%	F1: 0.52, Precision: 0.70, Recall: 0.48
[45]	5	500	Gradient agg. (decision tree)	Every round	99%	ASQ score: 98.7
[38]	4	10–20 until conv.	Federated model averaging (Docker/socket)	Each round	FER (Face) CNN-SVM: 71.64, Speech ensemble: 85.04	–
[40]	500	Up to 50	DHT-based model, P2P overlay	Every round	ResNet-34: 53%, ShuffleNet: 75.5%	F1: 0.48 (ResNet-34)
[43]	4 (hospitals)	5000	Personalized ensemble (async local/global)	Async after phase	EEG (Teacher): 85.8–88.7, ECG (Student): 80.4–85.4	Avg. Gmean reported
[44]	Multiple	(k-fold 10)	TF-based ensemble CNN, blockchain FL	After training cycle	99.19%	F1: 99.19%, Sensitivity: 99.99%, Specificity: 98.87%, TP: 99.45%
[31]	10	100–200	CAFed (async, DP noise), FedAvg	Async local updates	CAFed: 86.67%, Baseline CNN: 87.5%	F1 CAFed: 85.26%, F1 Baseline: 93.33%
[30]	8	Up to 400	FedAvg (IID/non-IID sim, DeepMood DNN)	Every round	Up to 86.95% (IID)	–

### 6.2. RQ2: How Diverse Is the Data for Predicting Mental Health Risks?

Understanding data diversity is central to the development of FL systems for mental health risk prediction. Unlike traditional centralised learning, these systems must learn from fragmented, multimodal, and inherently non-IID data distributed across heterogeneous devices and populations. The reviewed studies span physiological signals (EEG, ECG, and wearable sensors), neuroimaging, social media text, and smartphone interaction logs, each introducing different forms of variability in subjects, contexts, and hardware.

Two studies explicitly simulate non-IID client distributions using public datasets such as WESAD and MHEALTH, varying feature sets or sample size per client to approximate subject-level variation in wearable data [34,35]. These designs are consistent with broader FL work that stresses the importance of modelling subject-specific and multi-source heterogeneity in affective computing [17]. Other studies rely on naturally heterogeneous data from smartphones, social media, or embedded sensors, where class imbalance and behavioural variability create intrinsic non-IID conditions [27,29,41]. However, most of these works do not systematically quantify or mitigate distributional skew, even when instability arising from heterogeneous data is acknowledged as a limitation.

Multimodal and multi-platform data further increase diversity. Social media-based systems aggregate posts across platforms or languages, introducing linguistic and cultural variability that motivates domain adaptation or time-aware aggregation strategies [28,31,51,52]. Neurophysiological and neuroimaging applications operate on high-dimensional EEG, ECG, and MRI data, often combining hospital servers and wearable or fog-level devices [39,43,44], resembling broader proposals for blockchain-enabled and fairness-aware FL in neuroimaging [53,54]. A further layer of diversity arises from device-level heterogeneity: several studies report simulations or deployments across smartphones, wearables, fog nodes, and cloud servers yet rarely treat differences in compute, energy, or connectivity as first-class design constraints or fairness factors, despite evidence that device-aware scheduling can substantially improve both efficiency and equity [37,38,40,55].

Some works adopt multi-source or multi-view FL designs, combining keystroke dynamics, accelerometer readings, or clinical imaging to reflect fragmented mental health data streams [30,37,44]. These approaches align with recent multi-view FL frameworks that advocate for late fusion for asynchronous or noisy modalities [56]. However, across the corpus, the effects of data diversity on model performance, bias, and clinical usefulness are rarely examined explicitly. Fairness-aware analyses and systematic benchmarks for heterogeneous mental health data are rarely reported in the reviewed studies.

Across RQ2, the studies report diverse modalities, platforms, and devices, and a small subset explicitly simulate non-IID conditions or adopt hierarchical or multi-view FL to accommodate fragmented data. Detailed descriptions of dataset modalities, non-IID simulation strategies, and deployment contexts for each study are provided in Table 5.

**Table 5:** Data types, devices, public datasets, and diversity characterisations across studies. All studies use non-IID data across clients in a federated environment.

Study	Data Types	Edge Device	Public Dataset (If Any) (Accessed 23 December 2025)	Diversity Type/Heterogeneity	Privacy	Security
[34]	Physiological sensors: ECG, EDA, and EMG	Smartphone	WESAD (https://www.kaggle.com/datasets/mohamedasem318/wesad-full-dataset)	Feature and quantity imbalance (unequal volume and feature variation)		
[27]	Location, acceleration, and calls	Smartphone	No	Feature and quality imbalance		
[39]	MRI images	FL nodes (hospital datasets)	Alzheimer’s MRI (https://www.kaggle.com/code/ahmetesencan/alzheimer-mri-classification-using-cnn) and brain tumour	Modality imbalance across institutions		
[35]	Triaxial sensors (acc/gyro/magnetometer) and vital signs (HR and breathing rate)	Wearables/mobile health sensors	(MHEALTH, https://archive.ics.uci.edu/dataset/319/mhealth+dataset)	Multi-source, subject-level personalisation		Homomorphic Encryption
[36]	Activity recognition (walking, jogging, sitting, standing, and stairs)	Smartphone and wearables	WISDM	Multi-source, hierarchical FL		
[37]	Planned multimodal: images, radio samples, acoustics, and infrared	Residential IoT sensors/edge devices	No	Heterogeneous multi-source data	Differential privacy	Encryption
[40]	Computer vision (images) and NLP (audio); physical/behavioural	Edge devices/nodes (wearables, vehicles, gateways, and routers)	Google Speech; FEMNIST; (EUA, https://github.com/PhuLai/eua-dataset)	Multi-source data; heterogeneous edge environments	Differential privacy	Homomorphic encryption
[28]	Social media text (Reddit, Twitter, and Weibo)	FL nodes across platforms	GitHub collections: https://github.com/pg815/Depression_Detection_Using_Machine_Learning; RedditNet https://github.com/Diego-ds/RedditNet	Multilingual, multi-platform FL		Encryption
[42]	EEG data	Decentralised FL nodes	UCI/Code repo (https://github.com/BakerWade/Epileptic-Seizure-recognition)	Patient-specific FL models		
[41]	Physiological signals (HR, EDA, and posture) and facial expressions	Mobile edge (phones and wearables)	(Ford Challenge https://www.kaggle.com/c/stayalert); (SWELL)	Multi-source, subject-level imbalance		
[29]	Smartphone sensors (accelerometer, gravity, and battery)	Smartphones	No	Multi-source, real-world heterogeneity		
[45]	Multimodal ASD (ASQ, CSBS, PEDS, M-CHAT, STAT, EEG, and facial expressions)	Fog nodes/distributed lab compute	https://github.com/Abdullah-Lakhan/ASD-Code-and-Datasets/tree/mainASD repositories	Heterogeneous ASD datasets		AES encryption
[30]	Keystroke dynamics; accelerometer	Smartphones	Open source (https://github.com/RingBDStack/Fed_mood)	Multi-source mobile health data		
[38]	Facial expressions and speech signals	Smartphones, IoT, and on-board cameras/mics	No	Multimodal, subject variability, and device heterogeneity with varying data quantity and seven emotion classes		
[44]	MRI & rs-fMRI brain scans	Fog/edge nodes; blockchain-enabled	(ADNI https://adni.loni.usc.edu/data-samples/)	Modality heterogeneity (MRI vs. rs-fMRI), subject variability, and decentralised distribution		AES encryption
[43]	EEG & ECG signals	Wearable IoT, fog nodes, hospital servers, and on-board cameras/mics	TUH EEG (TUSZ https://service.tib.eu/ldmservice/dataset/tuh-eeg-seizure-corpus–tusz-)	Multi-biosignal processing, modality heterogeneity, subject variability, and decentralised distribution		
[31]	Social media text (Weibo posts)	Smartphones and cloud FL servers	No	Linguistic variability, user-behaviour heterogeneity, and asynchronous FL updates		

### 6.3. RQ3: What Privacy and Security Techniques Are Used?

Across the reviewed studies, the use of privacy and security techniques in FL varies substantially in both conceptual framing and practical implementation. While FL is widely adopted as a privacy-conscious machine learning framework, the assumption that decentralised data alone suffices for privacy preservation is increasingly challenged in contemporary literature. It has been shown that FL, even without access to raw data, remains vulnerable to privacy attacks such as membership inference [57,58] and gradient inversion [58,59], which can reconstruct sensitive input information from shared model updates.

As summarised in the Privacy and Security columns of Table 5, the majority of reviewed studies implement no formal privacy mechanism beyond the use of federated learning itself, leaving those entries blank. Only a small subset apply differential privacy or explicit encryption of model updates, and none implement secure aggregation or systematic access-control schemes.

The assumption that FL alone is sufficient for robust privacy protection is challenged both theoretically and empirically. Theoretically, studies have demonstrated that decentralisation does not prevent attacks such as membership inference or gradient inversion [57,58]. Empirically, most reviewed studies rely solely on FL’s decentralized architecture, without integrating additional safeguards such as differential privacy or secure aggregation, thereby exposing potential vulnerabilities [60].

Specifically, in the studies by Xu et al. [30], Alahmadi et al. [34], Zhang et al. [39], Chhikara et al. [38], Rashmi et al. [39], and Zhang et al. [36], federated learning is employed to prevent the direct exchange of raw data, but no additional privacy-enhancing mechanisms are applied. These studies fail to address how model parameters themselves are protected during or after transmission, nor do they quantify or bound the risk of privacy leakage. In contrast, only a limited subset of studies attempts to implement formal privacy-preserving techniques. Nurmi et al. [37] introduce local differential privacy (LDP) by adding perturbation to client-side updates prior to communication, while ref. [31] applies global differential privacy by injecting Gaussian noise into server-side aggregation. These measures align with the theoretical standards of ε-differential privacy (Dwork and Roth, 2014) [61], which provide quantifiable protection against re-identification [62]. However, the majority of studies that mention differential privacy, such as [35,40], do so only speculatively, framing it as a potential future direction, without actual implementation or evaluation. Similarly, cryptographic techniques such as homomorphic encryption, secret sharing, and secure multiparty computation (SMPC) are proposed in [35,37,40] but are never realised in practice due to known computational overheads (Zang et al., 2024) [63].

The treatment of communication security is notably inconsistent. The studies reported in [44,45] are the only ones to explicitly implement AES encryption for the protection of model parameter exchange, in line with best practices for transport-layer confidentiality. Ref. [28] references encryption in abstract terms but fails to specify the encryption algorithm or its point of integration. Most other studies, including studies [27,29,30,38,42,43], either entirely omit the mention of communication security entirely or implicitly assume that decentralised data suffice to mitigate associated risks. Given evidence that model updates transmitted in FL systems are subject to adversarial reconstruction (Xu et al., 2022) [64], this omission indicates a serious deficiency in the operational security of these studies. Notably, secure aggregation protocols such as those proposed by Mansouri et al. (2023) [65] are absent from all reviewed implementations, despite their increasing relevance in scalable FL deployment.

Access control and authentication mechanisms are largely absent across the corpus. Ref. [44] is the only article that implies a particular mechanism, which is a blockchain-based, double-range access-control mechanism that is intended to identify medical staff. It is important to note that the lack of access control in the rest of the studies is a severe gap in dealing with adversarial participation and unauthorised model contribution, as they are claimed to be real dangers in FL settings [66]. This is especially problematic in the healthcare sphere, where the sensitivity of the data and regulatory limitations demand strict authentication and auditability of participants.

Several studies also conflate architectural proposals with actual implementation. For example, Refs. [35,40] propose design-level architectures that include privacy-aware systems like homomorphic encryption and susceptible accumulation, without actually implementing empirical validation or performance metrics. In this regard, they should be considered rather conceptually limited in terms of value rather than ensuring privacy in a provable manner. Furthermore, studies like [31,37], which do operationalize the DP mechanisms, lack adequate investigation of the trade-offs between privacy budgets and model utility, in addition to not examining robustness in an adversarial setting. The performed critical assessment does not allow for the generalisation of the reliability and validity of the findings.

Overall, RQ3 shows that only a minority of the included studies implement formal mechanisms such as differential privacy or encryption, and none realises secure aggregation or systematic access control in deployed systems.

### 6.4. RQ4: What Challenges and Limitations Exist?

Reported limitations in the reviewed literature are consistent in nature, and we attribute them to methodological and operational limitations, especially in the areas of architectural scalability; broad, geographic deployability; system benchmarking; and clinical fidelity. Across studies, FL combined with cloud, fog, and edge infrastructures are typically described as exploratory, with varied attention to real-world applicability and diagnostic complexity [27,29,34,35,36,37,40,41,43,44,45].

Several studies use conventional cloud-based FL configurations using FedAvg aggregation without evaluating scalability under real conditions. Studies [27,29,34] identify sparse or no edge deployment. While communication cost and latency are mentioned, they are generally explained in terms of incomplete or even anecdotal data, such as mention of delays or resource strain, without mention of quantitative metrics, benchmarking, or comparative analysis.

Rashmi et al. [39], however, are explicit in noting the use of just two FL nodes to evaluate BrainCrossFed and caution against extrapolating scalability claims. Similarly, Zhang et al. [36] propose a hierarchy architecture across simulated clients but that lacks a real deployment on edge devices and resource-constrained hardware.

Although many studies invoke “edge–cloud” architectures, many implementations are limited to simulation. D.Y. Zhang et al. [41] empirically evaluate FL on Jetson devices and report energy consumption for stress and drowsiness detection, whereas other studies, such as those by Nurmi et al. [37] and Lakhan et al. [45], mention edge integration without reporting memory, power, or latency measurements. While Ching et al. (2024) [40] present peer-to-peer scalability via EC2 overlays; however, they do not investigate realistic conditions such as outdated connectivity, client churn, or skewed user behaviour simulation. Even in studies including physical components [27,29,30], experiments are usually limited and restricted with respect to their practice—e.g., in short-term pilots conducted with the authors themselves, there is a need for broader testing on devices under a range of operating conditions.

Evaluation practices are another area of acknowledged inconsistency. Several studies report high predictive performance results when in challenging, controlled settings; for example, Rashmi et al. [39] report 99.77% accuracy with BrainCrossFed, and Lakhan et al. [45] present similarly high metrics using CNN–LSTM hybrids. However, such evaluations often omit details about the dynamics of convergence under conditions of client heterogeneity, asynchronous client participation, or dropout. Xu et al. (2022) [30] note a 10 percent drop in accuracy for DeepMood under non-IID data but restrict training to high-resource GPUs, with mobile deployment described only as a direction for future work. Most studies, including [28,35,38], omit system-level benchmarks such as round duration, bandwidth usage, or inference footprint, despite operating in mobile or constrained environments. Only ref. [27] describes an adaptive scheduling mechanism that batches FL updates into three daily epochs (morning, evening, and night), archiving a reported 97 percent reduction in communication volume—though this was tested in a two-node pilot with limited generalisability.

Model–hardware alignment also poses practical challenges. Several studies use computationally intensive architectures (e.g., XLM-RoBERTa [28], DINOv2 [39], and CNNs [44]), without evaluating them for feasibility on edge devices. Although Refs. [34,41] report processing time or memory load for CNN-based models, they do not link these figures to device constraints such as heat dissipation, battery drain, or communication frequency. Baghersalimi et al. (2024) [43] offer a more hardware-aware evaluation by deploying quantised student models on Raspberry Pi Zero and Kendryte K210 devices but still note reduced generalisation performance compared to teacher networks and trade-offs between speed, accuracy, and hardware choice.

Finally, no study has implemented multi-label or comorbidity-aware learning frameworks—approaches that enable models to detect and classify despite multiple co-occurring conditions within individuals such as asthma, stroke, neurodegeneration, and work-related stress [39,40,41,44]. This is particularly relevant in mental health, where multimorbidity and diagnostic overlap are commonplace. The lack of such modelling contrasts with prior calls in the literature to prioritise multimorbidity-aware frameworks in healthcare AI [67] and continues to limit the real-world applicability of current FL systems.

Across RQ4, the most frequently reported limitations concern simulation-based evaluation, small client pools, scarce system-level metrics, limited alignment between models and deployment hardware, and the absence of comorbidity-aware learning frameworks [27,28,29,30,31,34,35,36,37,38,39,40,41,43,44,45].

Taken together, the findings across RQ1–RQ4 describe how current federated, cloud, fog, and edge approaches are implemented and evaluated in mental health applications; the following section interprets these patterns and considers their implications for clinical and infrastructural practice.

## 7. Discussion

The 17 reviewed studies reveal several recurring architectural patterns. Most systems still rely on cloud-centred FedAvg with limited edge deployment, while a smaller subset explores hierarchical, asynchronous, or decentralised overlays that better match heterogeneous devices. Common system limitations include small or simulated client pools, sparse reporting of system-level metrics (latency, energy, and bandwidth), and weak alignment between model complexity and edge hardware constraints. Deployment realism remains limited because many evaluations use software-only testbeds or short pilots, with few studies testing under realistic network variability, long-term use, or client churn. Together with narrow diagnostic coverage and minimal integration of formal privacy mechanisms, these issues create barriers to clinical adoption, where robustness, multimorbidity modelling, regulatory compliance, and end-to-end security are essential.

Across the 17 studies, only a small subset explicitly references formal data governance or regulatory frameworks; for example, Nurmi et al. [37] highlight GDPR and data-sovereignty considerations in their smart-home FL platform, whereas most other systems address privacy only at the algorithmic level, without specifying accountability for model updates, logging, or breach notification. Real-world deployment remains limited: a few studies report pilots on actual smartphones or embedded devices (e.g., [27,36,42]), but many evaluations are conducted in software-only or small-scale testbeds. Clinical validation and safety assessment are largely absent; none of the reviewed works conducts prospective trials in routine care, and only a minority explicitly involve clinicians or patients in system evaluation. These gaps in governance, regulation, deployment realism, and clinical validation currently limit the readiness of FL systems for widespread adoption in mental health services.

This review synthesises the results of 17 empirical studies that assessed the use of federated learning (FL), cloud, edge, and fog computing in relation to mental health applications. While the reviewed studies show increasing technical innovation, they also exhibit limited clinical realism, sparse evaluation of deployment, and weak interdisciplinary grounding. Overall, the field appears to be in a formative but fragmented stage, with diverse methodologies but no common frameworks for real-world scalability or clinical translation.

In order to ensure methodological rigour and comprehensive coverage, a systematic literature review (SLR) process was followed in this review. Studies were identified through structured searches across major databases (e.g., IEEE Xplore, PubMed, and Scopus) using predefined inclusion and exclusion criteria. Screening was conducted in multiple stages—title/abstract review, full-text assessment, and quality appraisal—guided by PRISMA principles. Data extraction focused on technical architectures, clinical domains, evaluation strategies, and interdisciplinary integration.

Federated learning remains the dominant architectural strategy underpinning decentralised mental health AI, particularly due to its privacy-preserving design and compatibility with distributed data sources (Dubey et al., 2025) [6]. The majority of studies implemented horizontal FL schemes with centralised aggregation—most often using FedAvg (e.g., Refs. [27,28,29,34]). Although algorithmic simplicity may account for this, few papers explored strategies for FL under asynchronous, decentralised, or hierarchical conditions. Zhang et al. [36] introduced a personalised multi-level FL framework tailored to heterogeneity in IoT environments, and Study 16applied decentralised FL for resource-constrained seizure detection. Li et al. (2023) [31] uniquely adopted asynchronous optimisation but lacked empirical benchmarking of the stability, energy cost, and fairness of the proposed model under partial client participation. These examples, while encouraging, represent isolated efforts within a broader landscape where convergence behaviour, non-IID data adaptation, and fairness-aware aggregation are largely underexplored.

Model selection across studies was, varied spanning CNNs (e.g., Refs. [39,44,45]), LSTM hybrids [45], and transformers [31], but the rationales for these choices in relation to real-world deployment constraints were rarely discussed. Only a subset of studies engaged meaningfully with edge-device limitations such as memory footprint, on-device latency, or inference efficiency. For instance, while Refs. [41,43] deployed FL on Jetson boards and wearable sensors, respectively, system-level performance metrics (e.g., update lag, communication cost, and thermal load) were inconsistently or qualitatively reported. These gaps suggest that FL models are often evaluated more for algorithmic behaviour than for full-stack feasibility.

In terms of infrastructure, cloud computing was often assumed but rarely interrogated in detail. Studies referencing cloud integration (e.g., Refs. [28,34,37]) mostly concerned themselves with storage or coordination functions, without much discussion of backend orchestration, the latency of services, or cost trade-offs under varying workloads. Fog computing, even though mentioned in several studies (e.g., Refs. [34,40,45]) was conceptualised as an intermediate layer between edges and the cloud, yet empirical evaluation of fog-layer performance (e.g., routing resilience, real-time responsiveness, or system failover) was absent. Edge deployment, while described in a number of papers (e.g., Refs. [35,36,41,43]), was more frequently simulated than realised, leaving questions around interoperability, energy management, and local processing still open.

The diagnostic landscape was also relatively narrow. The majority of studies focused on identification of depression or stress (e.g., Refs. [27,28,29,30,31]), while conditions such as autism [45], epilepsy [42,43], Alzheimer’s disease [39,44], and anxiety received comparatively limited attention. Even in studies considering multimodal data (e.g., Refs. [35,38]), cross-modal fusion was sparsely employed, and few of the frameworks took into account comorbidity-aware or multi-label architectures. This is a significant gap, considering the prevalence of diagnostic overlap in clinical mental health care. Prior reviews (e.g., Grataloup and Kurpicz-Briki, 2024 [10]; Khalil et al., 2024 [68]) similarly emphasized the importance of more nuanced, inclusive FL designs that reflect population-level heterogeneity.

Evaluation practices were widely varied. Predictive metrics such as accuracy and F1 score were universally reported, but system-level measures (e.g., inference delay, resource consumption, and fault tolerance) were usually omitted. Baghersalimi et al. [43] mentioned energy-aware constraints in wearable systems, without direct comparison of baseline architectures. Privacy-enhancing mechanisms were used occasionally: differential privacy was applied in [31], while Refs. [39,44] used blockchain for distributed authentication. However, the computational demands of the proposed models and their effects on utility were not empirically evaluated, contributing to the more general tendency in FL studies to overemphasize conceptual innovation over deployment maturity (Geyer et al., 2017 [69]).

Author-reported limitations repeat many of these issues. Sample sizes were frequently small or synthetic (e.g., Refs. [29,36,37,39]); deployment configurations were minimal, with several studies testing on only a handful of edge clients (e.g., Refs. [40,41]); and no included study conducted ablation analyses or user-centric validation under asynchronous or fault-prone settings. These patterns indicate that FL research in mental health remains mostly conceptual and preclinical, a consistent interpretation that is echoed by the recent literature (Khalil et al., 2024 [9]).

Taken together, the reviewed studies are indicative of both the promise and the incomplete architectures of FL-enabled mental health systems. While algorithmic creativity is evident, there are still lapses in deployment realism, system benchmarking, diagnostic inclusivity, diversity of design. In particular, very few studies have addressed the effects of edge–cloud coordination and fog-based buffering on downstream accuracy, fairness, and user latency in low-resource networks. Furthermore, no framework offers end-to-end modelling of resource-aware, privacy-preserving analytics across multiple devices, data types, and conditions.

Looking ahead, significant progress could depend on a number of strategic directions. First, integration between the design of the FL algorithm and system-level constraints such as intermittent connectivity, memory, and user behaviour would improve the viability of deployments. Second, the development of comorbidity-aware, multi-label models and multimodal fusion pipelines could enhance clinical relevance across diverse populations. Third, benchmarking frameworks should broaden to encompass latency, robustness, energy use, and fairness under asynchronous, non-IID, and low-participation regimes. Finally, cloud–fog–edge orchestration layers merit closer examination—not only in architectural diagrams but also in deployment trials that measure trade-offs across throughput, resilience, and patient-centric privacy.

These challenges, while significant, are addressable. They represent an evolving research frontier where federated mental health systems, if better aligned across technical and clinical domains, hold substantial potential to transform digital mental health and equitable care delivery.

Across the 17 studies, FL is predominantly implemented as cloud-centred horizontal FedAvg, with a central server coordinating updates from smartphones or IoT clients [27,29,30,31,35], while only a minority explore alternative architectures such as hierarchical or decentralised schemes [36,39,41,43,44]. Overall, standard cloud-based FedAvg is more frequently used than hierarchical, decentralised, or edge-native schemes, which appear in only a small subset of the reviewed work.

The observed cloud–edge deployment choices have direct implications for latency, energy efficiency, scalability, and privacy. As FL moves from conceptual frameworks to real-world implementations, these design decisions will determine whether systems are performant and ethically robust in practice. A single study demonstrates a fully decentralised, cloud-free deployment across hospital devices [43], which aligns with recent proposals for zero-trust, peer-based FL in healthcare [70] but also raises open questions about global coordination, interpretability, and clinical accountability.

Overall, the cloud–edge integration strategies reported in Section 6 reveal a broad design space, from private clouds and public platforms to embedded hardware, yet there is little empirical evaluation of end-to-end latency, energy use, and fault tolerance.

The evaluation patterns observed in RQ1 are consistent with wider concerns that FL lacks agreed-upon protocols for assessing utility, efficiency, and resilience in distributed environments. While some papers provide detailed predictive metrics, many neglect latency, scalability, communication cost, and robust non-IID testing, leaving the current evidence base short of the multi-layered validation needed to judge deployment readiness in decentralised mental health settings. This gap contrasts with broader federated learning research that stresses the need to measure and stress test systems under realistic heterogeneity [28,50].

## 8. Conclusions

This systematic review synthesised 17 empirical studies that integrate federated learning with cloud, fog, and edge computing for mental health applications, indicating growing interest in decentralised, privacy-preserving analytics. The evidence, however, depicts a technically innovative but pre-deployment field: most systems rely on cloud-centred FedAvg with small or simulated client pools, focus predominantly on depression or stress, and rarely implement or benchmark formal privacy and security mechanisms in realistic settings.

To move towards clinically useful and operationally robust systems, future work needs to co-design FL algorithms with resource-aware cloud–fog–edge infrastructure; expand to comorbidity-aware and multimodal models that reflect the heterogeneity of real populations; and routinely report system-level metrics such as latency, robustness, and energy use. Equally important is the embedding of explicit data governance and regulatory considerations, together with prospective user-centred evaluations that assess safety, usability, and effectiveness in routine services.

With stronger alignment between algorithmic design, systems engineering, clinical practice, and ethics, federated learning has the potential to evolve from promising prototypes into scalable, trustworthy tools for mental health care in diverse and dynamically changing environments.

## Data Availability

The original contributions presented in this study are included in the article/Supplementary Material. Further inquiries can be directed to the corresponding author.

## Supplementary Materials

The following supporting information can be downloaded at: https://www.mdpi.com/article/10.3390/s26010229/s1, PRISMA Checklist Table S1: PRISMA 2020 checklist; PRISMA Abstract Checklist Table S2: PRISMA abstract checklist.

## Appendix A

### Appendix A.1

**Table A1:** Excluded studies (n = 14, score < 7/10).

DOI (Link)	First Author, Year	Q1	Q2	Q3	Q4	Q5	Total	Exclusion Rationale
E1 https://doi.org/10.1109/JSAC.2022.3213323	Wang 2022	1	1	2	1	1	6	Generic blockchain incentive mechanism for hierarchical FL; no mental health task or outcomes.
E2 https://doi.org/10.1145/3603166.3632559	Casella 2024	1	2	1	1	1	6	Vertical FL aggregation method; not applied to mental health; no edge/cloud mental health deployment.
E3 https://doi.org/10.1145/3594739.3610693	Saisho 2023	1	1	1	0	1	4	Ubiquitous computing demo paper; FL and edge discussed but not a full mental health AI system with robust evaluation; a poster contribution for UbiComp/ISWC ’23 Adjunct conference.
E4 https://doi.org/10.1007/s10878-022-00939-x	Gokulakrishnan 2022	1	2	2	0	1	6	Focuses on physical security around hospitals, not mental health; FL privacy not evaluated as mental health safeguard.
E5 https://doi.org/10.1007/s00521-021-06434-4	Khowaja 2023	1	1	1	0	1	4	Generic IoMT healthcare framework; FL and privacy mostly conceptual; no concrete mental health AI experiment.
E6 https://doi.org/10.1038/s41746-020-0244-4	Perez-Pozuelo 2024	1	0	0	0	1	2	Narrative review of sleep-health data; no federated learning implementation or edge/fog/cloud FL deployment.
E7 https://doi.org/10.1007/s11063-023-11182-8	Melanson 2023	1	1	1	1	1	5	Secure multi-party computation for general activity recognition, not mental health; FL not the focus; no cloud/edge mental health system.
E8 https://doi.org/10.1007/s11301-024-00441-0	Hornik 2024	1	0	2	0	0	3	Fog computing AI for marketing management; no FL, no mental-health AI, and no relevant privacy mechanisms for clinical contexts.
E9 https://doi.org/10.1109/JIOT.2023.3299736	Jiang 2024	1	2	1	1	1	6	Stress monitoring with clustered FL but aims framed as general stress/wearable monitoring rather than mental health AI; edge integration and privacy partially specified.
E10 https://doi.org/10.1007/s11042-021-10990-1	Kalamaras 2021	0	0	0	0	1	1	Graph-based visualisation of sensitive medical data; no FL, no edge/fog/cloud FL, and no mental health AI experiment.
E11 https://doi.org/10.1016/j.jksuci.2024.101940	Sorour 2021	2	0	0	1	2	5	Centralised deep learning for Alzheimer’s classification; no federated learning; no edge/fog/cloud deployment; privacy not evaluated in federated setting.
E12 https://doi.org/10.1016/j.jad.2024.10.027	Kuang 2024	2	1	1	1	1	6	Adolescent depression prediction uses FL but limited detail on edge/cloud deployment and privacy mechanisms; system-level evaluation not aligned with RQs.
E13 https://doi.org/10.1016/j.compbiomed.2024.108344	Gonzalez 2024	1	0	1	0	1	3	Multipurpose mobile health service architecture; no implemented FL pipeline or mental health-specific empirical evaluation.
E14https://doi.org/10.1007/s10489-024-05796-1	Sen 2024	1	0	0	0	1	2	Occupational ergonomics application; no FL implementation, no mental-health AI, and no edge/fog/cloud FL deployment.

### Appendix A.2

**Table A2:** Included studies (primary studies) (n = 17, score ≥ 7/10).

Citation	First Author, Year	Q1	Q2	Q3	Q4	Q5	Total	Status
[34]	Alahmadi et al., 2024	2	2	2	1	2	9	Included
[27]	Suruliraj & Orji, 2022	2	2	2	1	2	9	Included
[39]	Rashmi et al., 2023	2	2	2	1	2	9	Included
[35]	Shaik et al., 2022	2	2	2	1	2	9	Included
[36]	C. Zhang et al., 2024	2	2	2	1	2	9	Included
[37]	Nurmi et al., 2023	2	2	2	2	2	10	Included
[40]	Ching et al., 2024	2	2	2	1	2	9	Included
[28]	Liu, 2024	2	2	2	1	2	9	Included
[42]	Suryakala et al., 2024	2	2	2	1	2	9	Included
[41]	D. Y. Zhang et al., 2021	2	2	2	1	2	9	Included
[29]	Tabassum et al., 2023	2	2	2	1	2	9	Included
[45]	Lakhan et al., 2023	2	2	2	1	2	9	Included
[30]	Xu et al., 2022	2	2	2	1	2	9	Included
[38]	Chhikara et al., 2021	2	2	2	1	2	9	Included
[44]	Mandawkar & Diwan, 2024	2	2	2	1	2	9	Included
[43]	Baghersalimi et al., 2024	2	2	2	1	2	9	Included
[31]	Li et al., 2023	2	2	2	2	2	10	Included
